# Electrical and Electrochemical Monitoring of Nucleic Acid Amplification

**DOI:** 10.3389/fbioe.2015.00029

**Published:** 2015-03-05

**Authors:** Tatsuro Goda, Miyuki Tabata, Yuji Miyahara

**Affiliations:** ^1^Institute of Biomaterials and Bioengineering, Tokyo Medical and Dental University (TMDU), Tokyo, Japan

**Keywords:** PCR, isothermal amplification, electrical biosensor, pH monitoring, field-effect transistor

## Abstract

Nucleic acid amplification is a gold standard technique for analyzing a tiny amount of nucleotides in molecular biology, clinical diagnostics, food safety, and environmental testing. Electrical and electrochemical monitoring of the amplification process draws attention over conventional optical methods because of the amenability toward point-of-care applications as there is a growing demand for nucleic acid sensing in situations outside the laboratory. A number of electrical and electrochemical techniques coupled with various amplification methods including isothermal amplification have been reported in the last 10 years. In this review, we highlight recent developments in the electrical and electrochemical monitoring of nucleic acid amplification.

## Introduction

Nucleic acid amplification is a standard procedure for detecting and sequencing a small amount of DNA and RNA in life science research and molecular diagnostics. The objects include nuclear DNA, mitochondrial DNA (mtDNA), cytosolic DNA, messenger RNA (mRNA), ribosomal RNA (rRNA), and a series of non-coding RNAs (ncRNAs). Amplification of RNA is conducted after reverse transcription (RT) reaction where a complementary DNA (cDNA) is synthesized by RNA template by reverse transcriptase. The quantification of mRNA in coupled with the RT process is a common way of evaluating the degree of gene expression in molecular biology and clinical research. Recently, the determination of ncRNA level has gained popularity since ncRNA turned out to regulate gene expression in various ways in living organisms. Nowadays, microRNA (miRNA), a class of short ncRNAs, which is enveloped in extracellular vesicles or is protected by protein in body fluids such as serum, lymph, cerebrospinal fluid, urine, and saliva is of particular interests as a potent biomarker to predict the progression of cancer or tumor for early diagnosis (Calin and Croce, 2006). The combination of immunoassays with polymerase chain reaction (PCR) extends the applicability of nucleic acid amplification toward sensitive and specific recognition using antigen–antibody interaction. This method termed immuno-PCR benefits not only from the specificity of antibody to target antigen such as protein or small molecule but also the sensitivity of PCR (Sano et al., 1992). Similarly, a direct PCR from nucleic acid aptamer, which is an alternative molecular recognition element capable of specifically bind to a target analyte, allows a sensitive detection of a various kind of biomolecules (Zhou et al., 2007; Lee et al., 2009; Csordas et al., 2010; Zhang et al., 2011; Huang et al., 2013; Wang et al., 2013c).

PCR is a standard technique for DNA amplification. The process is composed of thermal cycles, in which designed forward and reverse DNA primers attach to a segment for copying in a DNA template through the Watson–Crick complementary base-pairing at 60°C, the attached primers are extended by a DNA polymerase in the presence of deoxynucleotide triphosphates (dNTPs) as a building block at 72°C, the copied DNA duplex is heat-denatured into single strand at 95°C, and the next cycle starts with the twice number of the DNA segment for copy at 60°C. Hence, the copied number of DNA segment ideally increases by twofold after a cycle of the thermal treatment. PCR is useful as an end-point assay to test the existence of target sequence of DNA in a sample. On the other hand, quantitative information of initial template in a sample cannot be determined by PCR. To aid this issue, real-time PCR or quantitative PCR (qPCR), has been invented. The method detects the accumulation of amplicon after each thermal cycle in real time so that the initial concentration of target sequence can be determined by a comparison with the amplification kinetics between the target and an internal control with known copy number. The running of qPCR is commonly monitored by optical method by using a custom fluorescent probe. The probe undergoes conformational switching or binds to double-stranded DNA (dsDNA) products to emit fluorescence as the DNA copying proceeds in an exponential way. Moreover, the high efficacy of PCR, which can amplify target from single molecule as well as massively parallel measurement led by advances in nanofabrication and microfluidics has realized a new strategy for nucleic acid quantification, namely Digital PCR (Baker, 2012). This is a counting approach based on binary nature of amplified signals. Digital PCR conducts real-time or conventional PCR in each individual well after partitioning of a dilute sample into hundreds or even millions of aliquots. In accordance with the Poisson distribution law, each reaction chamber contains zero or one copies of the sequence of interest. The positive signal for amplification is resulted only from the wells containing single target molecule and no signal is observed from the blank wells. By counting the both fractions after PCR, therefore, the absolute number of target molecule can be determined without the requirement of reference standards or internal controls.

## Amplification Techniques

**Figure 1:**
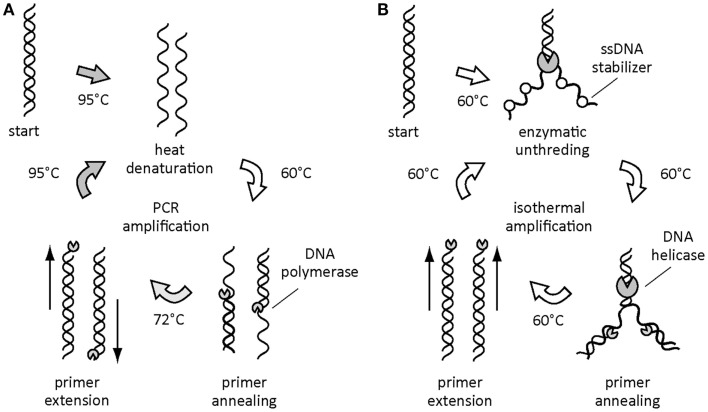
**Thermal and isothermal DNA amplifications**. DNA amplification by PCR during thermocycling process **(A)**. Helicase dependent amplification as an example of the isothermal DNA amplifications **(B)**.

As nucleic acid amplification by PCR becomes more general at the bench in a laboratory, there is a growing demand for developing a simpler method without the requirement of thermocycling process toward point-of-care testing. Because, in general, thermal cyclers are costly and large in size, consuming a large amount of energy, which cannot be afforded by portable batteries. To this end, a wide variety of isothermal amplification method has been developed (Figure 1; Table 1) (Pioch et al., 2008; Craw and Balachandran, 2012; Wang et al., 2012; Yan et al., 2014). Some of the technologies were invented by learning from natural DNA synthesis by polymerase and assisting proteins *in vivo* under isothermal conditions (Craw and Balachandran, 2012).

**Table 1:** **Summary of current and emerging technologies for isothermal DNA amplification**.

Entry	Amp. Temp.	Amp. type	Provider	Comment
HDA	60–65°C	Exponential	NEW ENGLAND BioLabs, UK	HDA requires helicase for unthreading dsDNA template, as well as two primers and polymerase same as PCR
RPA	37–42°C	Exponential	TwistDX, UK	RPA employs a recombinase for inserting primers with dsDNA template and ssDNA-binding protein for stabilizing the displaced strands. Reaction occurs under a reduced temperature
LAMP	60–65°C	Exponential	Eiken Chemical, Japan	LAMP is strand displacement-type amplification, but suffers from restriction in primer design by requiring at least four primers
RCA	30–60°C	Liner	GE Healthcare, UK, etc.	RCA requires a circular primer and φ29 polymerase. Mild temperature is suitable for point-of-care testing. The RCA-derivatives amplify target exponentially
PG-RCA	30–60°C	Exponential	–	
BRCA	30°C	Exponential	–	
DRCA	30°C	Exponential	–	

Helicase-dependent amplification (HDA) employs DNA helicase to unthread dsDNA instead of conventional heat denaturation step at 95°C in PCR (Vincent et al., 2004). The separated nucleotides by helicase are protected by single-stranded DNA (ssDNA)-binding protein, allowing the binding of primers to initiate elongation by polymerase at a constant temperature of 60–65°C without any thermal steps. The newly synthesized dsDNA products are then used as substrate for the next round of the chain reaction, which result in exponential amplification of target sequence as a function of incubation period.

Recombinase polymerase amplification (RPA) introduces a recombinase for inserting two opposing primers with a template in duplex DNA, a ssDNA-binding protein for stabilizing the displaced strands of DNA and for preventing the primers from being displaced, and strand-displacing polymerase for synthesizing DNA where the primers has bound to the template DNA (Piepenburg et al., 2006). Importantly, all the RPA processes proceed continuously under physiological temperature of 37–42°C with no other sample treatment to execute the amplification.

Loop-mediated isothermal amplification (LAMP) uses four different primers specifically designed to recognize six distinct regions on the target DNA (Notomi et al., 2000). The primers form stem-loop conformations with self-priming ability to initiate the LAMP cycling. The LAMP process is performed by a strand-displacing DNA polymerase at a constant temperature of 60–65°C.

Rolling circle amplification (RCA) employs a circular ssDNA primer via ligation and initiates extension from an ssDNA template annealed to the circular primer by a strand-displacing φ29 bacteriophage DNA polymerase (Fire and Xu, 1995). After completing DNA synthesis in the primer loop, the strand displacement ability continues to synthesize a new strand by displacing the previously formed dsDNA and eventually produces a long ssDNA amplicon with repeated sequence of the original target. The whole RCA reactions proceed under isothermal conditions (30–60°C). Primer generation-rolling circle amplification (PG-RCA) is an advanced mode of RCA, which amplifies target sequence in an exponential manner by applying nicking reaction to the long ssDNA amplicon followed by initiating another RCA reaction from the fragmented amplicon as a new template (Murakami et al., 2009). PG-RCA uses a circular primer designed to contain a domain for nicking enzyme in addition to a complementary sequence to the target. For highly sensitive determination of small nucleic acids, branched RCA (BRCA) by introduction of a second primer complementary to the RCA product was developed (Lizardi et al., 1998; Cheng et al., 2009). BRCA can improve the reaction efficiency compared with conventional linear RCA by producing large amounts of ssDNA and dsDNA with various lengths. Dumbbell probe-mediated RCA (DRCA) is another form of enhanced RCA methods (Zhou et al., 2010). The dumbbell probe is composed of a target binding domain, an indicator binding domain, and a loop domain. The binding of target to binding domain trigger DRCA reaction in the presence of ligase and polymerase.

Furthermore, there are several reports on a new amplification reaction based on a target recognition-triggered autonomous cascade reaction of DNA replication and nicking process in molecular machineries under isothermal condition (Wang et al., 2013c, 2014; Freage et al., 2014).

## Detection Methods

### Conventional optical methods

A number of optical methods have been applied for detecting nucleic acid amplification. Blotting of an agarose gel after DNA electrophoresis is a traditional way for identifying a PCR amplicon. For real-time PCR, a standard technique is a fluorescence-based detection in which the fluorescence switches on as a result of either generation of target duplex and/or conformational change of custom probes during nucleic acid amplification. Fluorescence-based intercalator or groove binder (e.g., SYBR^®^ green) increases its fluorescence intensity on forming a complex with dsDNA as the amplification progresses. TaqMan^®^ probe is an engineered short oligonucleotide complementary to amplification domain and possesses a pair of fluorescence and quencher in the molecule. The quenched probe is enzymatically digested by a DNA polymerase with exonuclease activity during PCR, resulting in the recovery of fluorescence intensity because of the physical separation of fluorophore with the quencher. In DNA sequencing by double strand synthesis, Illmina^®^ DNA sequencer distinguish each base by encoded deoxyribonucleotide triphosphate (dNTP) with four types of fluorescences at local colonies of DNA cluster produced by a custom DNA amplification on a chip.

Reaction byproduct such as proton (H^+^) and pyrophosphate ion (PPi) from the synthesis of complementary chain by DNA polymerase also serves as an indicator for progression of the reaction. Many optical assays have been developed for detecting PCR as a color change in a sample solution as a result of complex formation between an inorganic cation (e.g., Mo^6+^ and Mg^2+^) and PPi (Burns et al., 1991; Gibson et al., 1997; Mori et al., 2001). Sellamuthu et al. (2015) developed a phenanthroimidazole-based dizinc(II) fluorescent probe as a sensitive and specific indicator for PPi in PCR and pyrosequencing. Alternatively, chemiluminescence has been applied for quantification of nucleic acid amplification products (Martin et al., 1995). The detection of PPi as a product during primer-directed polymerase extension has been applied for a commercial pyrosequencer with advanced features of low cost and high throughput (Ronaghi et al., 1998). The signal transduction is based on the quantitative conversion of PPi to adenosine triphosphate (ATP) by sulfurylase, and the subsequent production of visible light by firefly luciferase.

### Electrical methods

Electrical and electrochemical monitoring of nucleic acid amplification requires no optical assistance so that the system can be simplified, downsized, and integrated into a small chip with the aid of complementary metal oxide semiconductor (CMOS)-compatible fabrication process, leading to the production of a scalable high-throughput analysis system in point-of-care applications. Advantageously, all the electrical systems described in this article are direct sensing of synthesized DNA amplicon or the side product so that they are label-free technologies without the requirement of tag or reporter molecules. In DNA sequencing, Sakata and Miyahara (2006) developed a semiconductor-based technique in which an ion-sensitive field-effect transistor (ISFET) directly senses local changes in the electrical charge or pH as a result of extension reaction at the end of probe DNA hybridized with target DNA in an offset manner on the gate area by DNA polymerase on addition with one of four dNTPs in a stepwise manner. Further, Rothberg et al. (2011) successfully developed an integrated ISFET system, which resulted in the commercial production of massively parallel high-throughput DNA sequencer. The semiconductor device acquires data for sequencing by sensing the protons produced by the template-directed DNA polymerase synthesis on microbeads in microfluidic channels.

**Figure 2:**
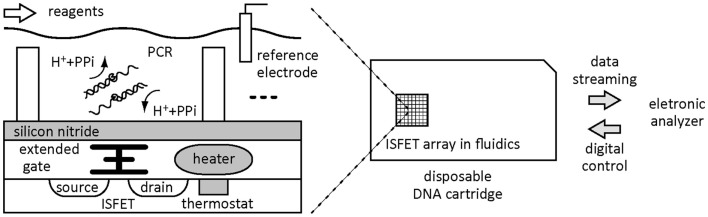
**A high-throughput electrical monitoring of DNA amplification by directly detecting protons generated by extension reaction in each well using the integrated ISFET array system**.

Toumazou et al. (2013) reported a method of electrical monitoring of qPCR and isothermal amplification using integrated ISFETs in a chip. The technique is based on the sensing of pH changes as a result of proton production during DNA synthesis in real time (Figure 2). The strategy is analogous to that used by the semiconductor-based Ion Torrent DNA sequencer (Rothberg et al., 2011). That is, ISFET enables a direct detection of protons produced by extension reaction by DNA polymerase. An integrated circuit with on-chip ISFETs, thermal cycler, and control circuitry is in contact with a reaction mix with low buffering capacity in wells to measure local pH changes proportional to the amount of synthesized DNA. The pH-based technique, in principle, requires no custom primers such as TaqMan probes and molecular beacons. The limit of detection was 10 copies of target in a 2-μl well. Nucleic acid amplification by RCA on a silicon nanowire transistor was detected as a change in drain-source current in real time (Gao et al., 2013). Following hybridization of a target DNA with a probe DNA immobilized on the nanowire and a free RCA primer in a sandwich manner, RCA reaction takes place to synthesize a long ssDNA as a polyanion, leading to changes in the electronic state of the silicon nanowire. The limit of detection was as low as 50 aM target DNA in an initial solution. Liu and Yobas (2013) developed an electrical system for real-time PCR monitoring by ionic current rectification in a conical nanofluidic diode. Synthesized DNA amplicon electrostatically adsorbs on the nanopipette tips that modulates the ionic rectification. Since the adsorbed mass of DNA amplicons in the glass nanocapillaries is dependent on the number of cycles in real-time PCR, the system can distinguish the mass concentration of the target DNA above 2.5 ng/μl.

### Electrochemical methods

**Figure 3:**
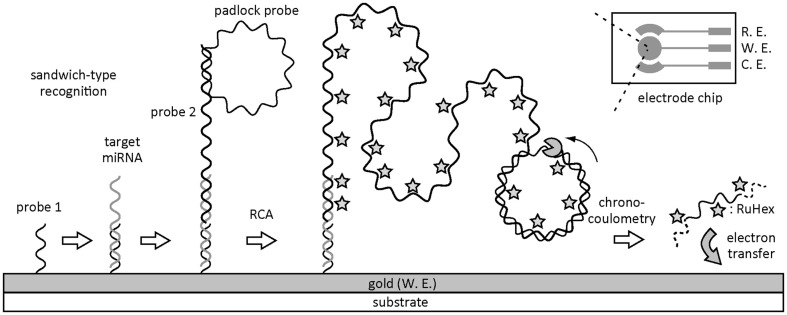
**Schematic illustration of electrochemical detection of miRNA after isothermal RCA of reporter probe on a solid surface**.

Detection of the tiny amount of nucleotide with the aid of electrochemical signal amplification has been reported in various manners such as amperometry, voltammetry, and AC-impedance as summarized in the literature (Luo and Hsing, 2009; Patterson et al., 2013). The techniques are categorized into two systems, that is, solid phase amplification and solution phase amplification. One representative solid phase amplification employs target-induced primer extension to interact with an electrochemical indicator to gain the signal from molecular recognition events such as hybridization and aptamer/protein binding (Chen et al., 2012; Cheng et al., 2012; Zhu et al., 2013). Nucleic acid amplification in combination with immunogenic recognition can be performed on a solid surface by taking care of steric factors that control the surface hybridization of amplified sequences (Del Giallo et al., 2005; Mix et al., 2009; He et al., 2010; Ferguson et al., 2011). Yao et al. (2014) successfully detected tumorgenesis-related miRNAs from serum samples with high sensitivity by hybridization-based miRNA capturing and subsequent isothermal RCA reaction for reporter sequence for amplifying the redox signal in chronocoulometry (Figure 3). Similarly, molecular beacon structure-mediated RCA was conducted for ultrasensitive electrochemical detection of nucleic acids (Ji et al., 2012; Wang et al., 2013b). Li et al. combined RCA with DNAzyme-guided polymerization of aniline for ultrasensitive electrochemical detection of DNA (Hou et al., 2014). An *in situ* hybridization chain reaction of DNA on a gold nanoparticle following immounosensing achieved ultrasensitive detection of protein biomarker (Zhou et al., 2012). Several groups in China reported signal amplification by isothermal circular strand-displacement polymerization induced by target binding to a DNA-based hairpin switch (Fu et al., 2013; Wang et al., 2013a).

**Figure 4:**
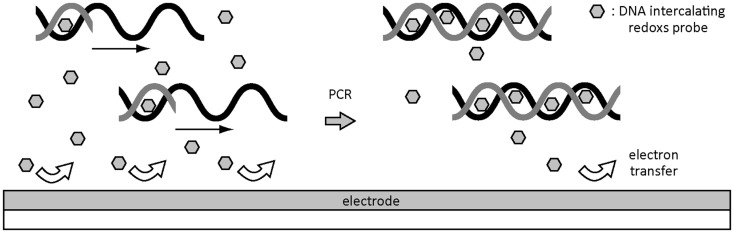
**Principle of electrochemical monitoring of DNA amplification during solution-phase real-time PCR using a custom redox probe with DNA intercalating ability**.

Solution-phase amplification is straightforward to conventional PCR process and is beneficial to the high efficiency of amplification process by liberating from the steric hindrance by nucleotides and polymerase (Fang et al., 2009; Ferguson et al., 2009; Mix et al., 2012). Real-time electrochemical monitoring of solution-phase amplification has been achieved by developing a DNA intercalating redox probe (Figure 4) (Defever et al., 2009, 2011). The osmium-based custom electroactive intercalator shows a strong and specific binding ability to dsDNA amplicon, no inhibition to the PCR process, a chemical stability under thermocycling, and a mild redox potential that fits in the potential window of conventional electrodes. The redox current is attenuated by increasing the number of PCR cycles because the redox intercalator become less active to an electron exchange following the complex formation with DNA amplicon compared with its free counterpart due to steric and diffusional constraints.

Isothermal amplification process is less prone to reduced redox activity of indicator due to the lack of thermocycling. Several researchers employed LAMP in solution phase in a disposable microfluidic channels for sensitive electrochemical DNA identification toward point-of-care testing (Ahmed et al., 2010; Hsieh et al., 2012; Safavieh et al., 2014). Lee et al. (2013) demonstrated isothermal amplification by PG-RCA and real-time electrochemical monitoring of DNA amplicon by methylene blue as an intercalating redox probe. HDA has been employed for a real-time isothermal amplification of nucleic acid to monitor a decrease in the electrochemical current response of a reporter DNA intercalating redox probe (Kivlehan et al., 2011). Wang et al. conducted capturing of target DNA followed by RCA reaction on a magnetic nanoparticle surface in solution phase for sensitive identification of DNA (Ding et al., 2013). Isothermal DNA amplification using G-quadruplex reporter molecule in solution phase has been developed (Nie et al., 2014). Hsing et al. successfully employed isothermal circular strand displacement polymerization without immobilizing a target-capturing custom hairpin probe on an electrode surface (Xuan et al., 2012).

## Conclusion and Outlook

This review is focused on recent technologies for electrical and electrochemical monitoring of nucleic acid amplification motivated by a growing demand for decentralized nucleic acid sensing in many applications. Substituting the optical setting for qPCR into electrical or electrochemical systems is advantageous in regards to simplification and miniaturization of the device. Among them, the semiconductor-based technology is amenable to low-cost production of portable device that allows massively parallel analysis of nucleic acid amplification for point-of-care testing. Further, the developments of thermocycling-free amplification process play a key role for realizing a ubiquitous nucleic acid screening and sensing in real world applications. The development of affordable and accessible point-of-care devices for nucleic acid sensing would find numerous potential applications. The first example would be the use to prevent outbreak of infectious diseases caused by deadly viruses such as Ebola virus, Crimean-Congo hemorrhagic fever virus, human immunodeficiency virus (HIV), and influenza viruses by performing early diagnosis and therapy. *In situ* analysis of food and water contaminations would also help to avoid foodborne illness caused by pathogenic bacteria, viruses, or parasites. Portable, wireless, and inexpensive devices for nucleic acid sensing may realize personalized healthcare through tailoring of interventions to individual patients at affordable cost.

## Conflict of Interest Statement

The authors declare that the research was conducted in the absence of any commercial or financial relationships that could be construed as a potential conflict of interest.
